# Supplementary figures and images for: Ubiquitin-interacting motifs of ataxin-3 regulate its polyglutamine toxicity through Hsc70-4-dependent aggregation
Source: eLife. 2020 Sep 21;9:e60742. doi: 10.7554/eLife.60742 (PMC7505662; doi:10.7554/eLife.60742)

Figure 2D

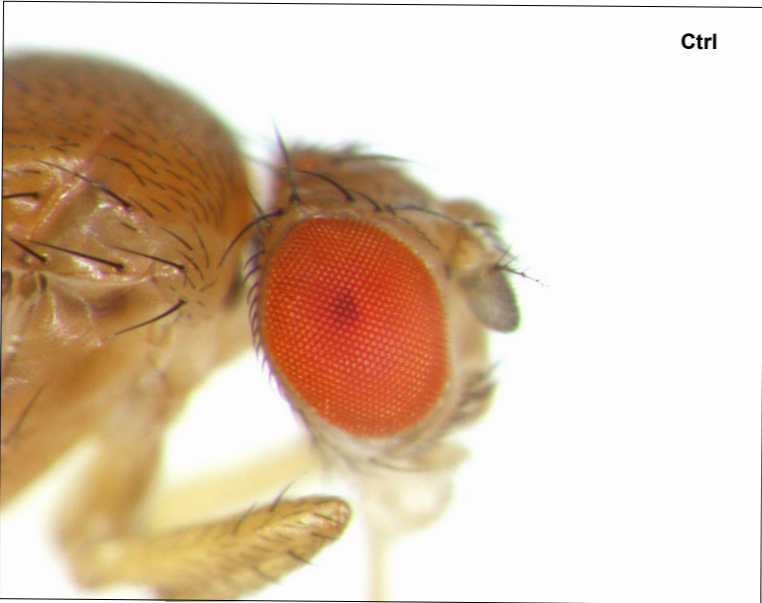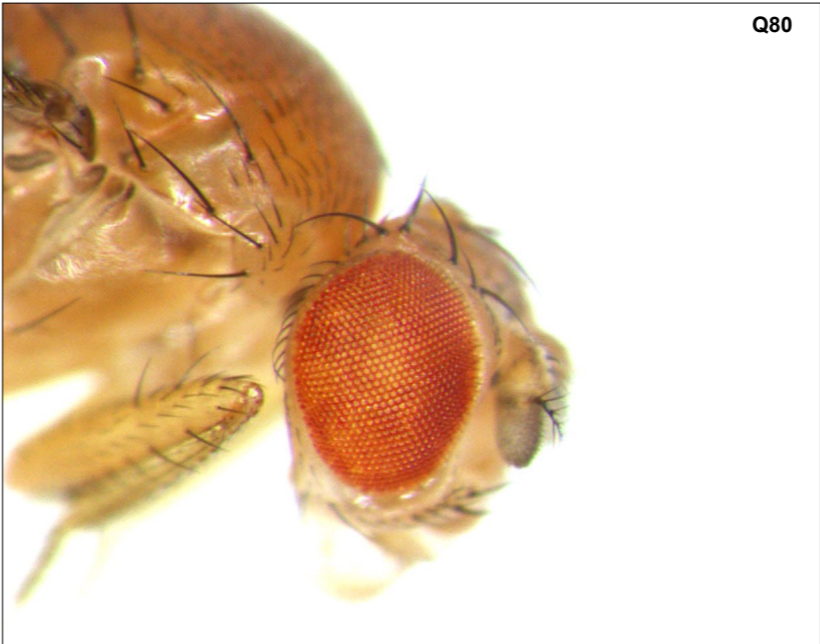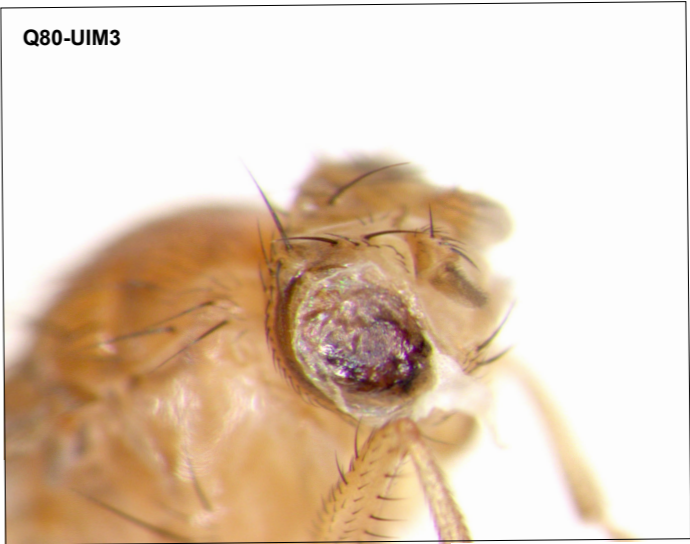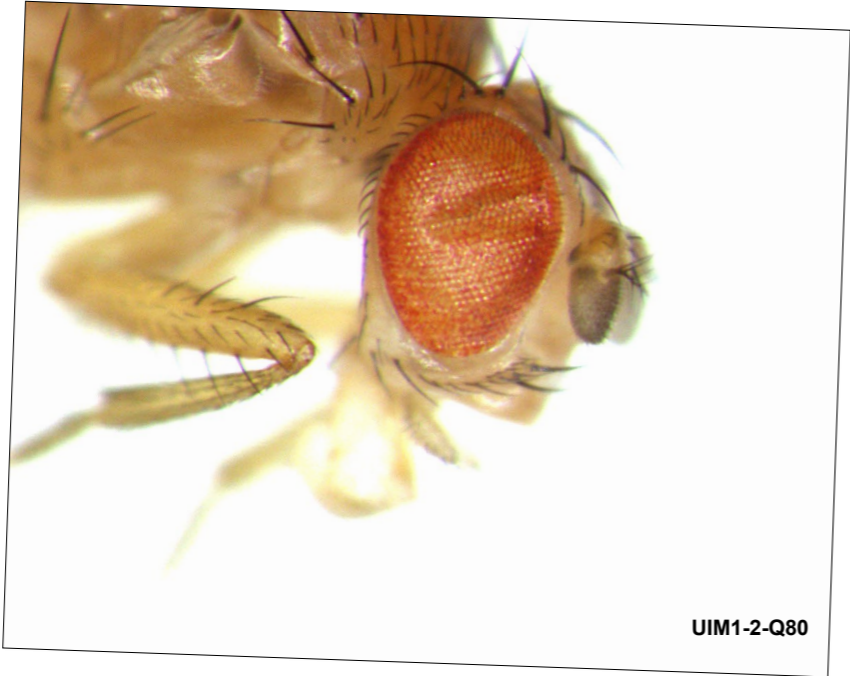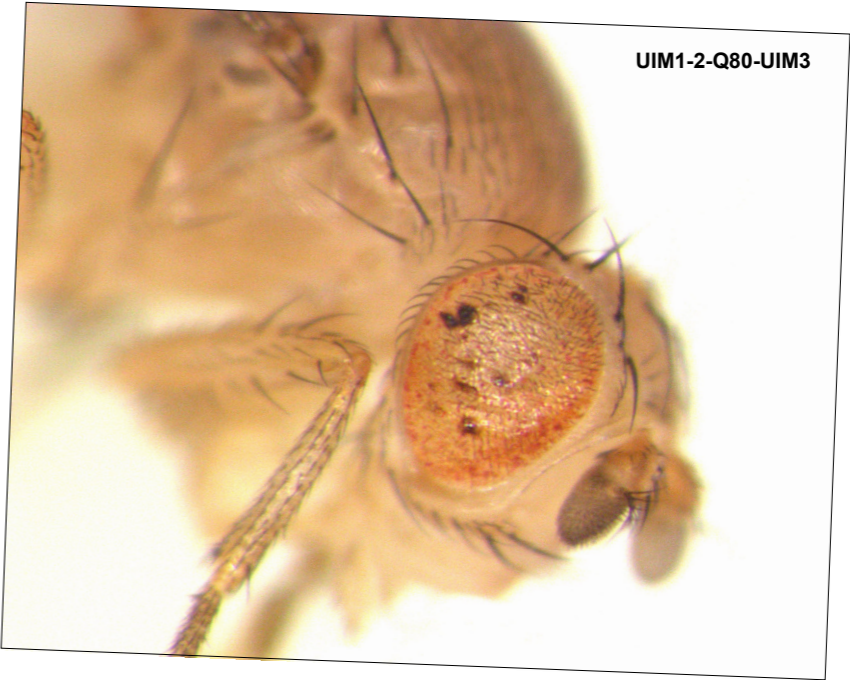

Supplement: Figure 2—source data 2. [file elife-60742-fig2-data2.pdf.zip › Figure 2 Source Data 2.pdf]

**Figure 4A**

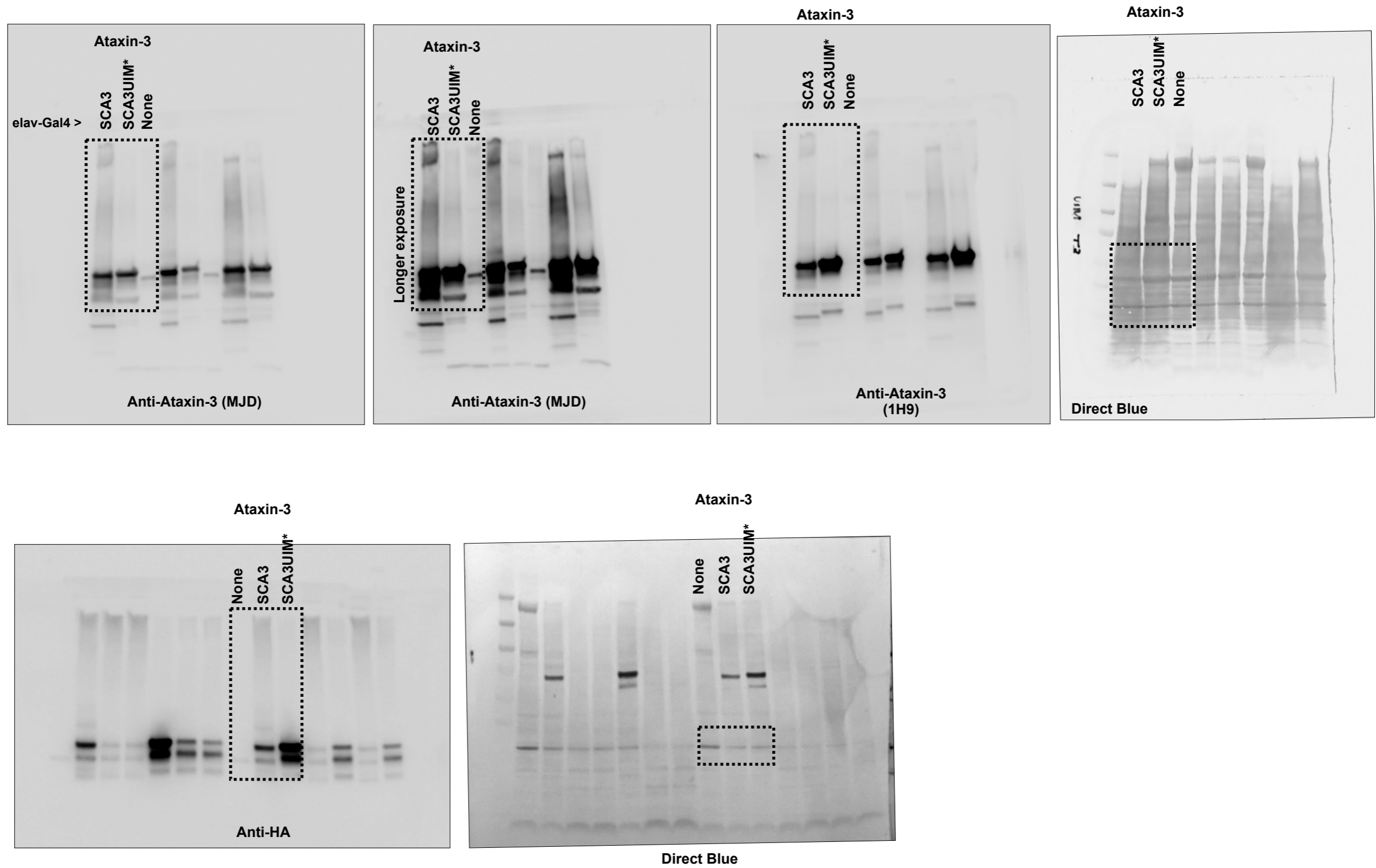

Figure 4B

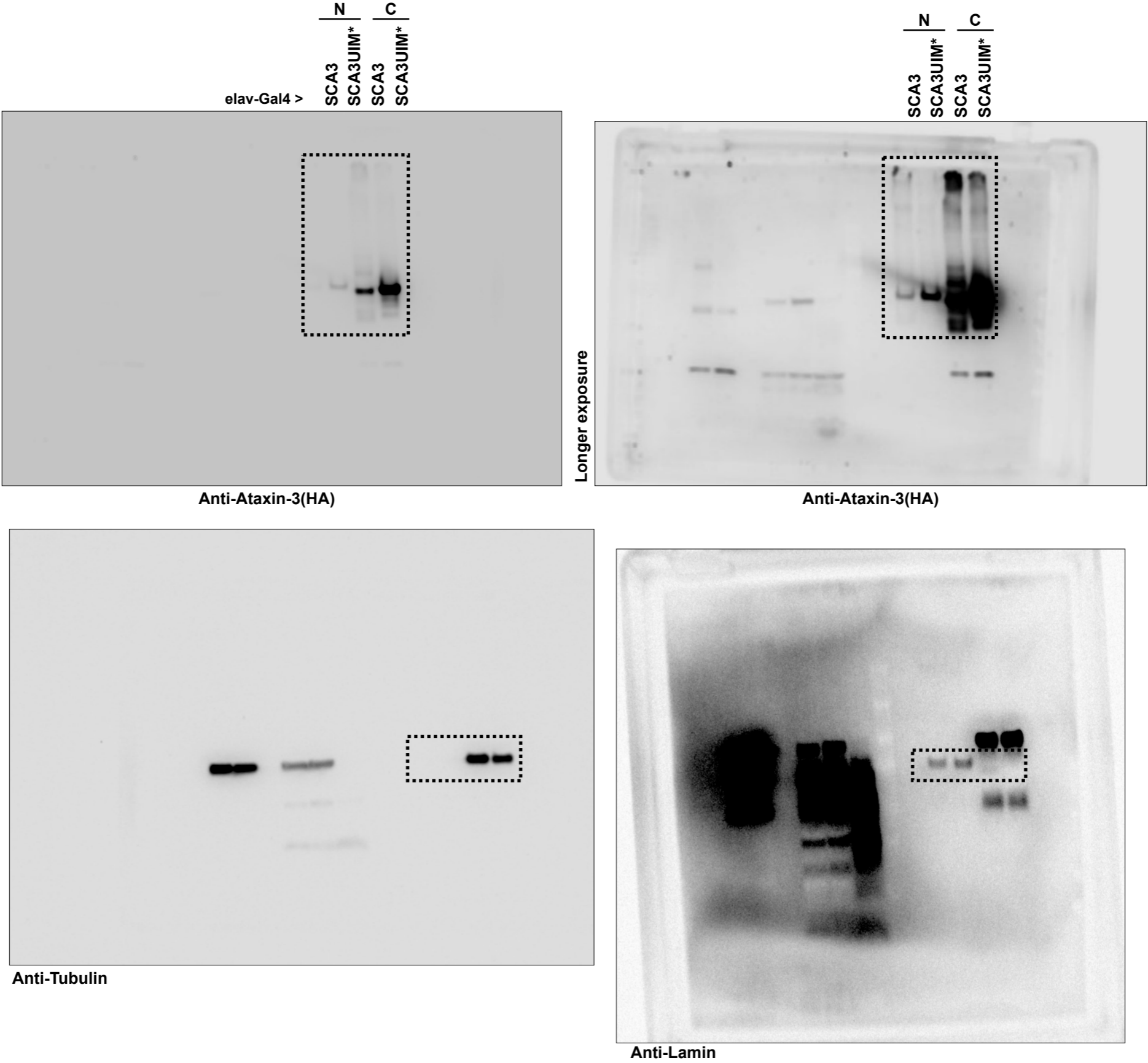

Figure 4C

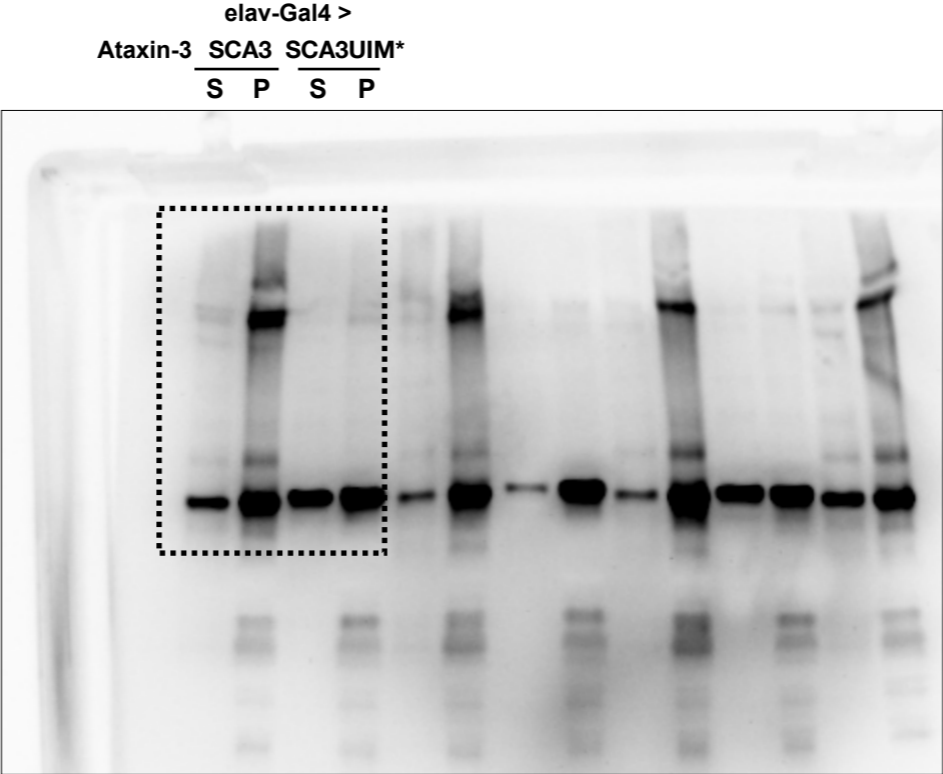

Anti-Ataxin-3 (1H9)

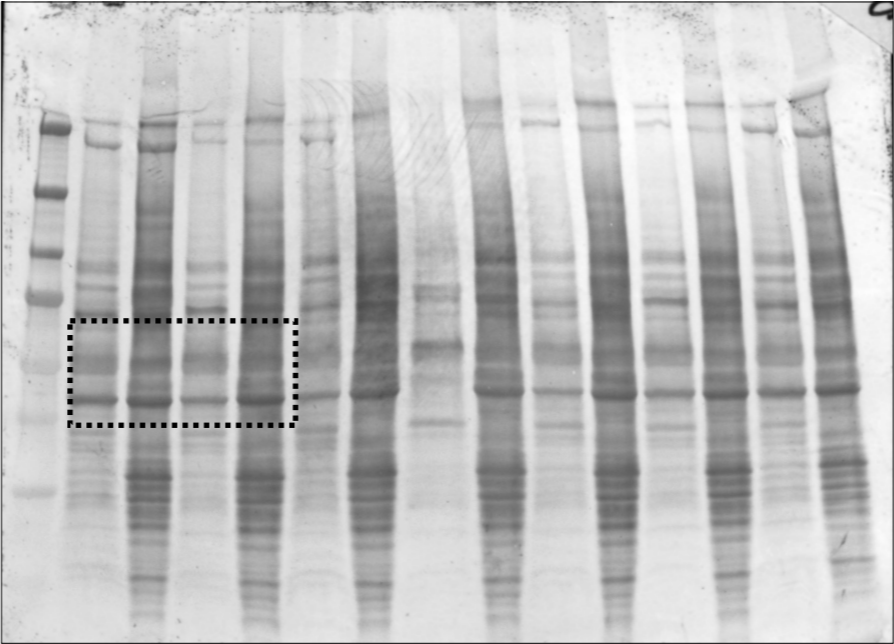

Direct Blue

**Figure 4D**

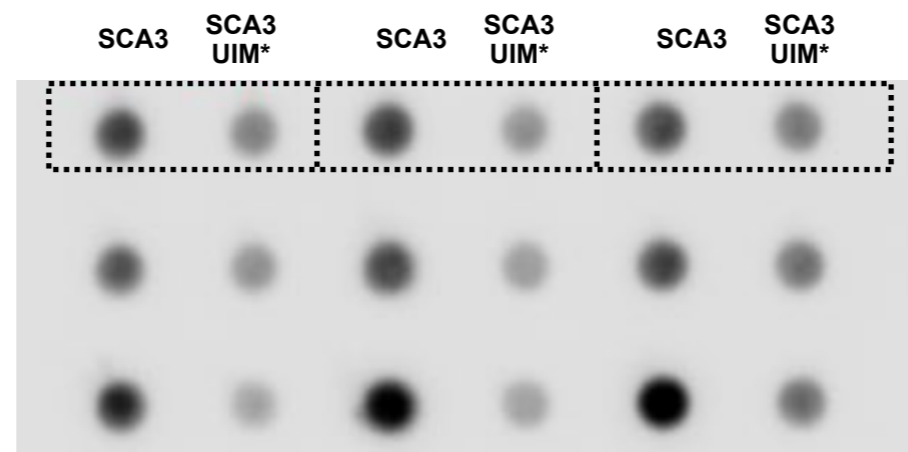

Supplement: Figure 4—source data 2. [file elife-60742-fig4-data2.pdf.zip › Figure4SourceData2.pdf]

**Figure 5A**

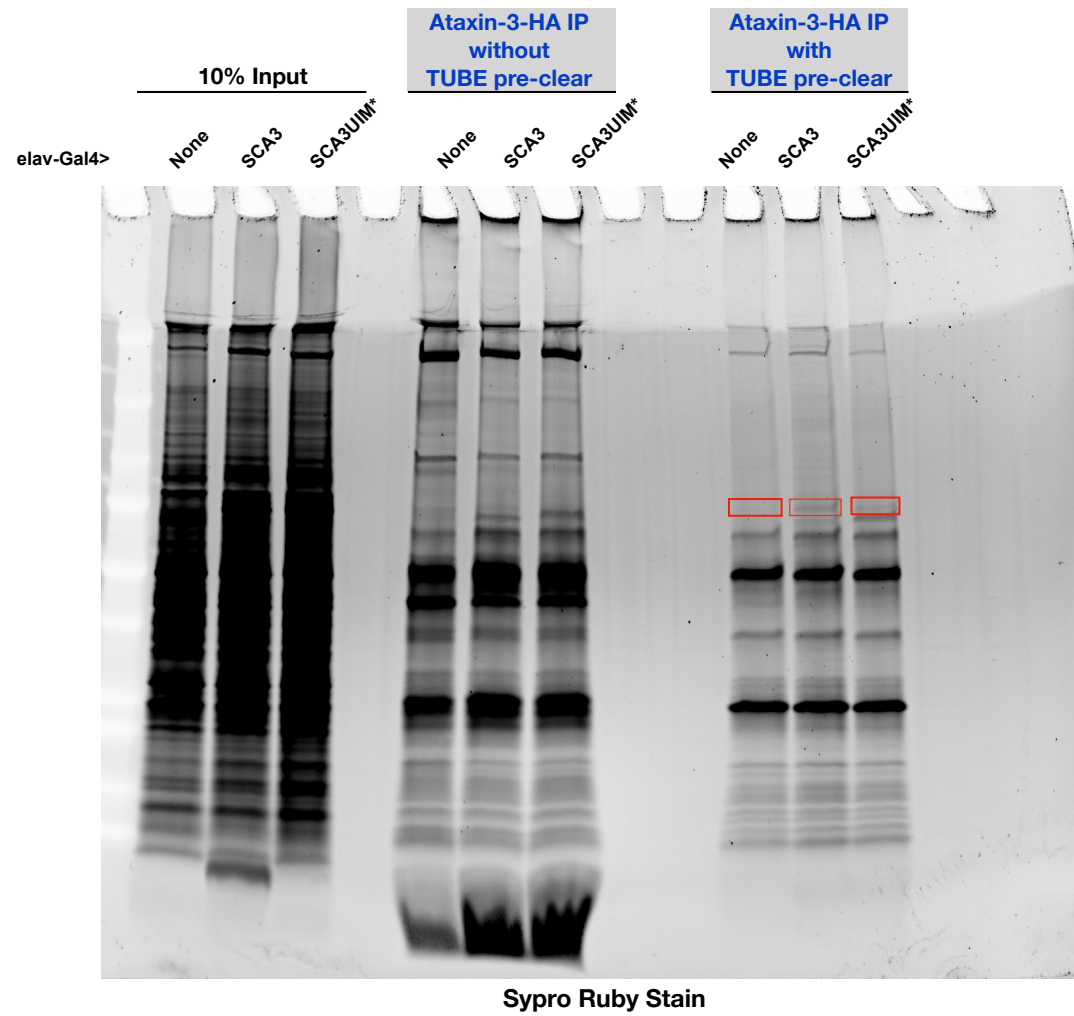

Figure 5B

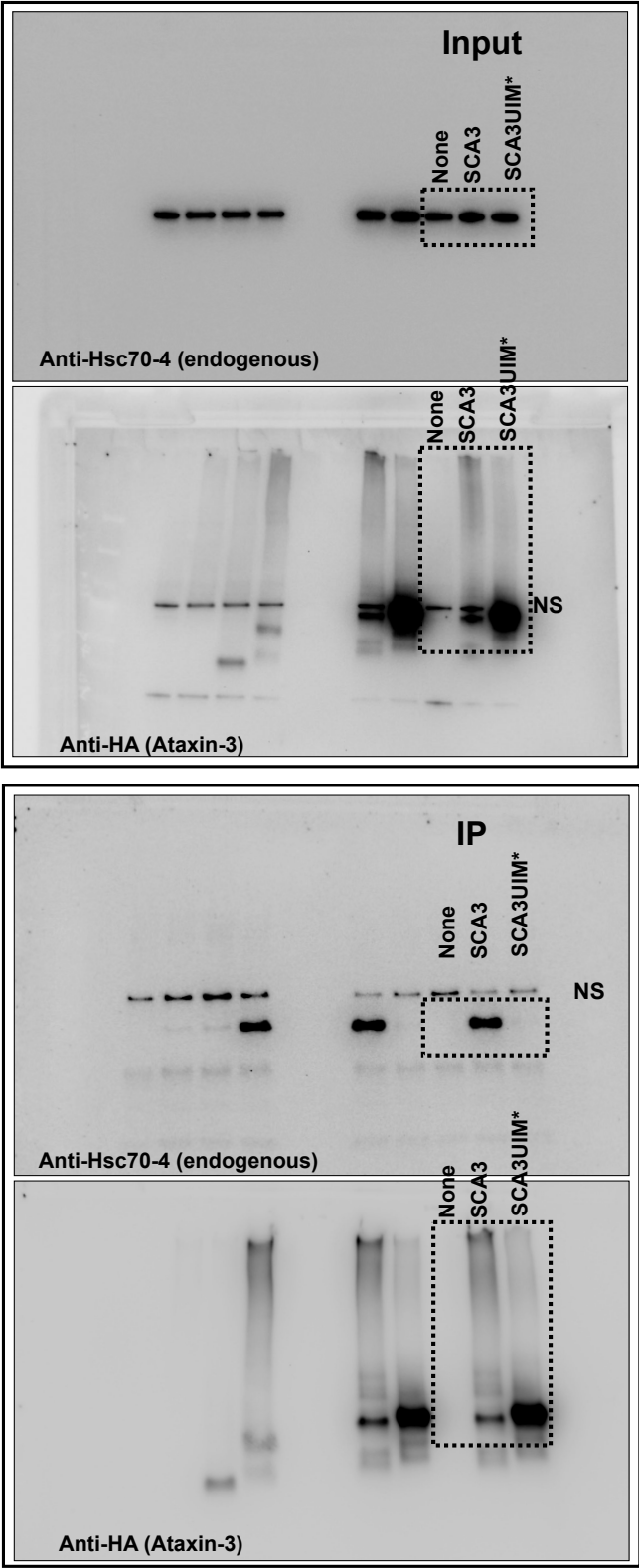

**Figure 5C**

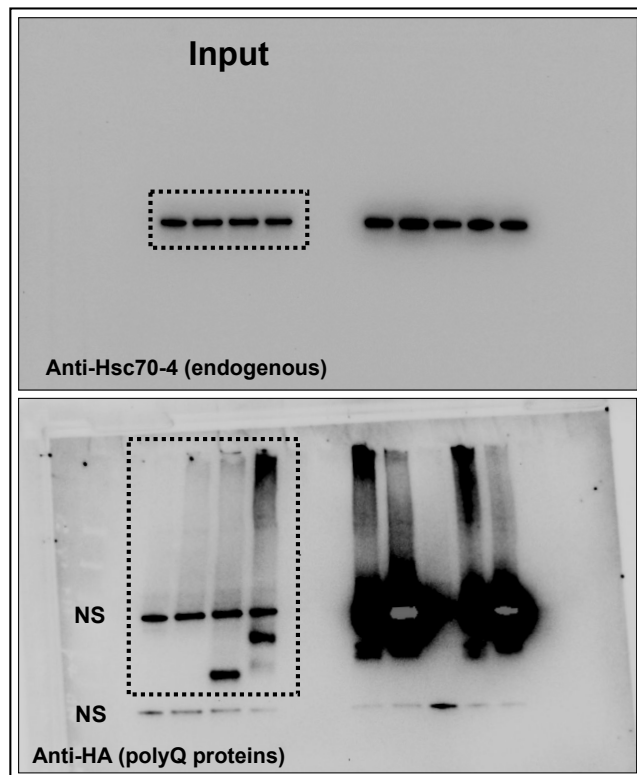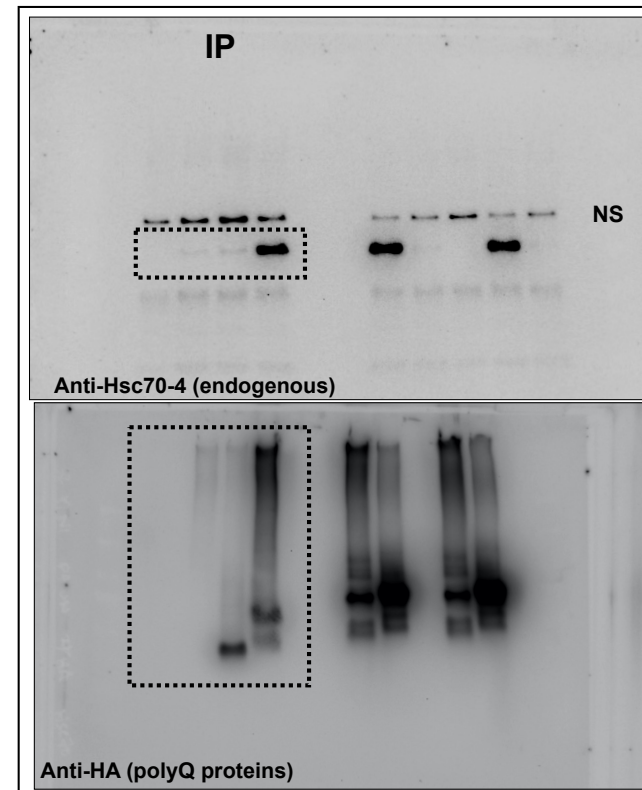

Figure 5D

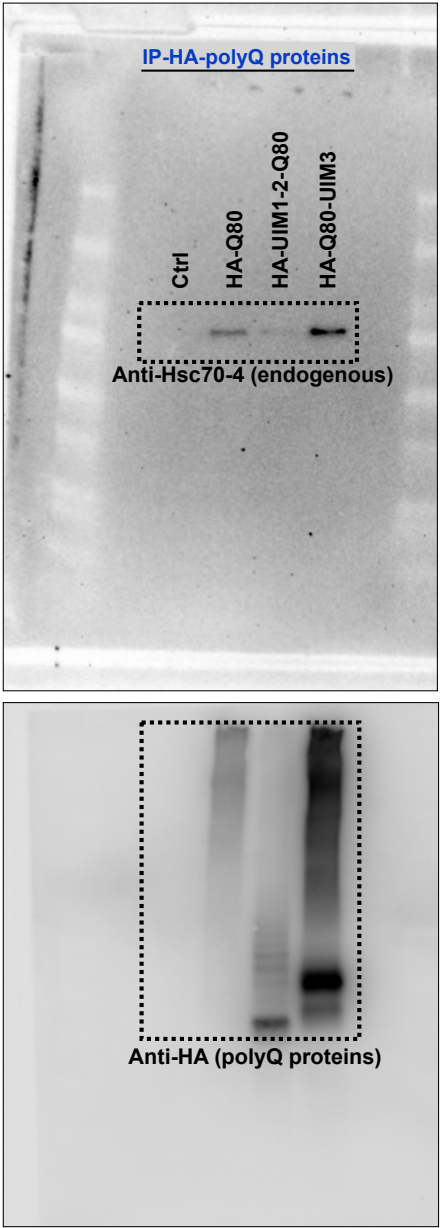

Supplement: Figure 5—source data 1. [file elife-60742-fig5-data1.pdf.zip › Figure5SourceData.pdf]

Figure 6A

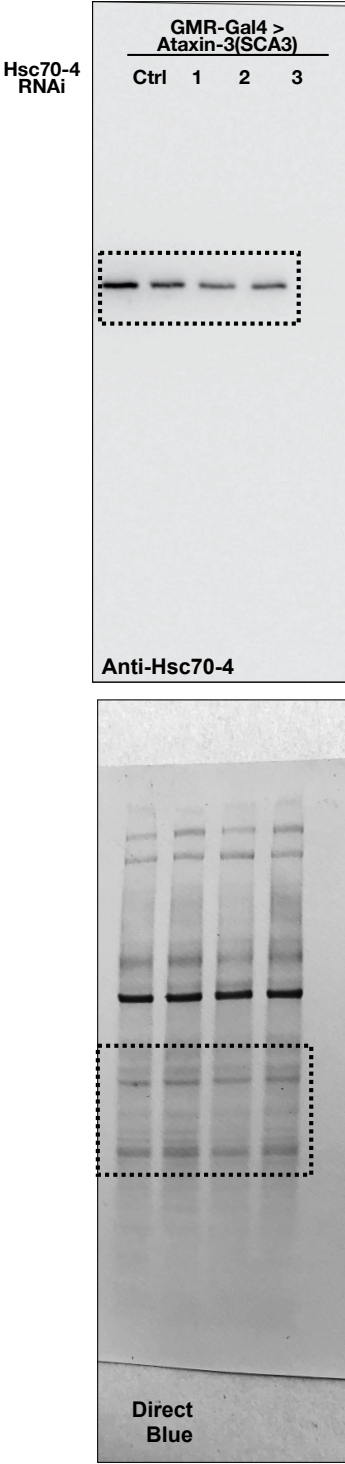

Figure 6B

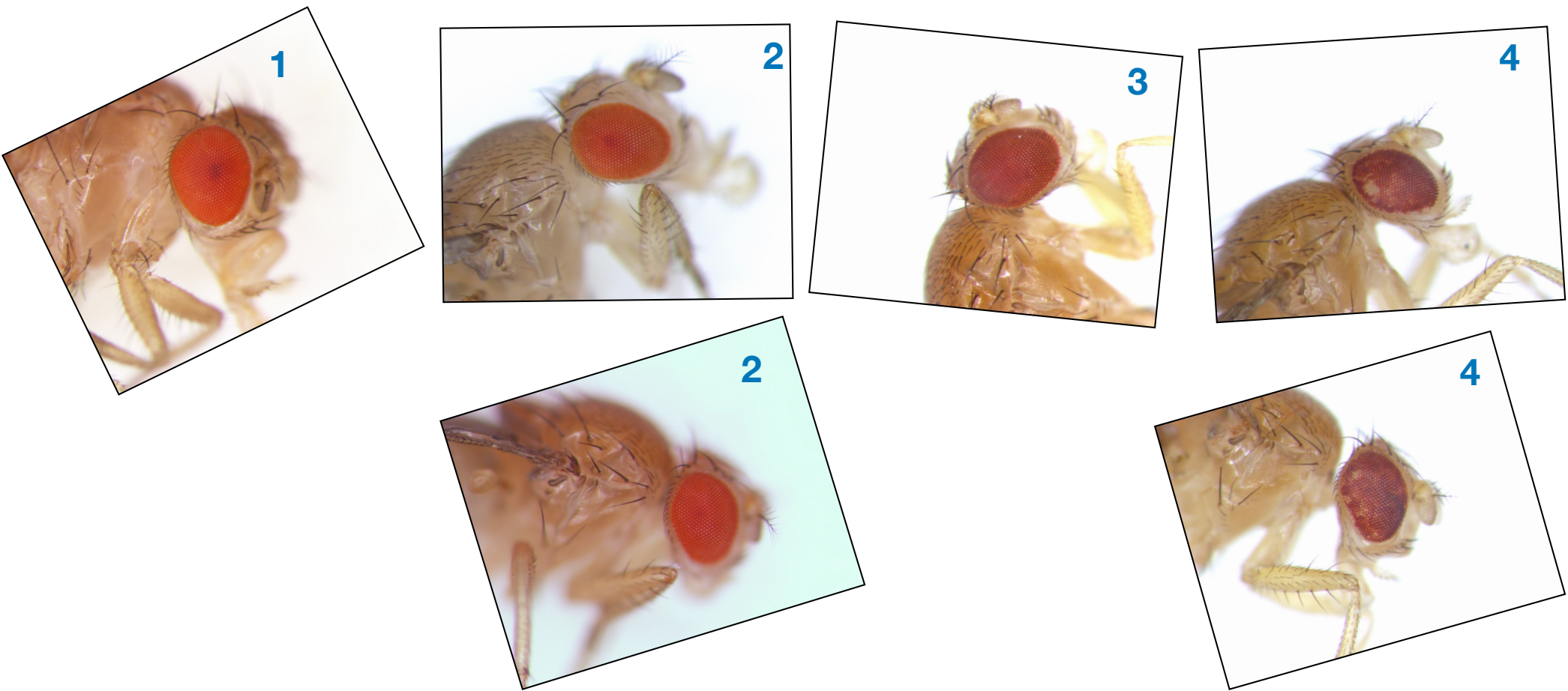

Figure 6D

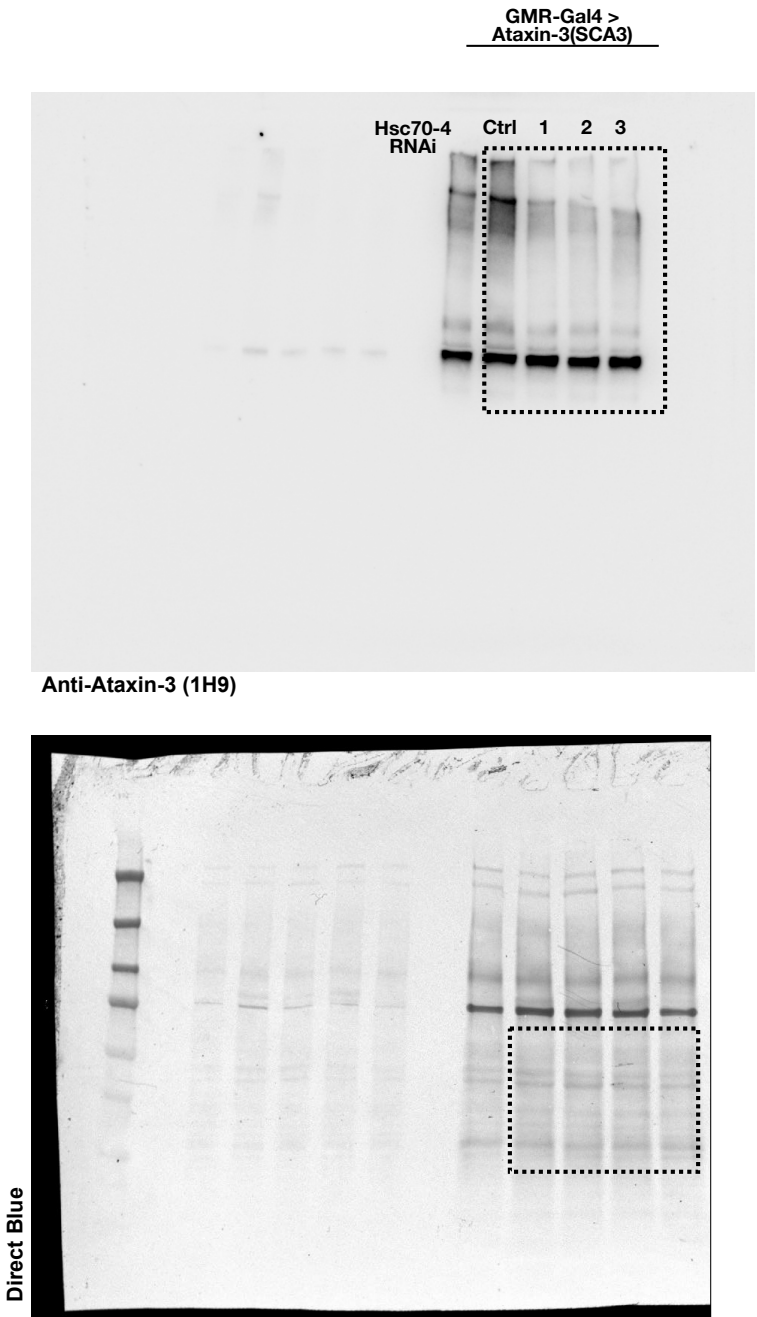

Figure 6E

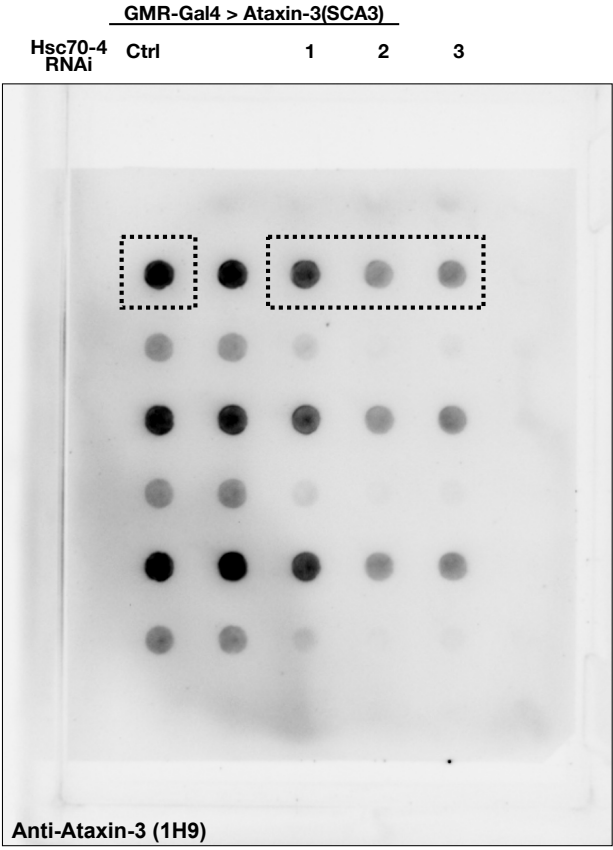

Supplement: Figure 6—source data 2. [file elife-60742-fig6-data2.pdf.zip › Figure6SourceData.pdf]

**Figure 7A**

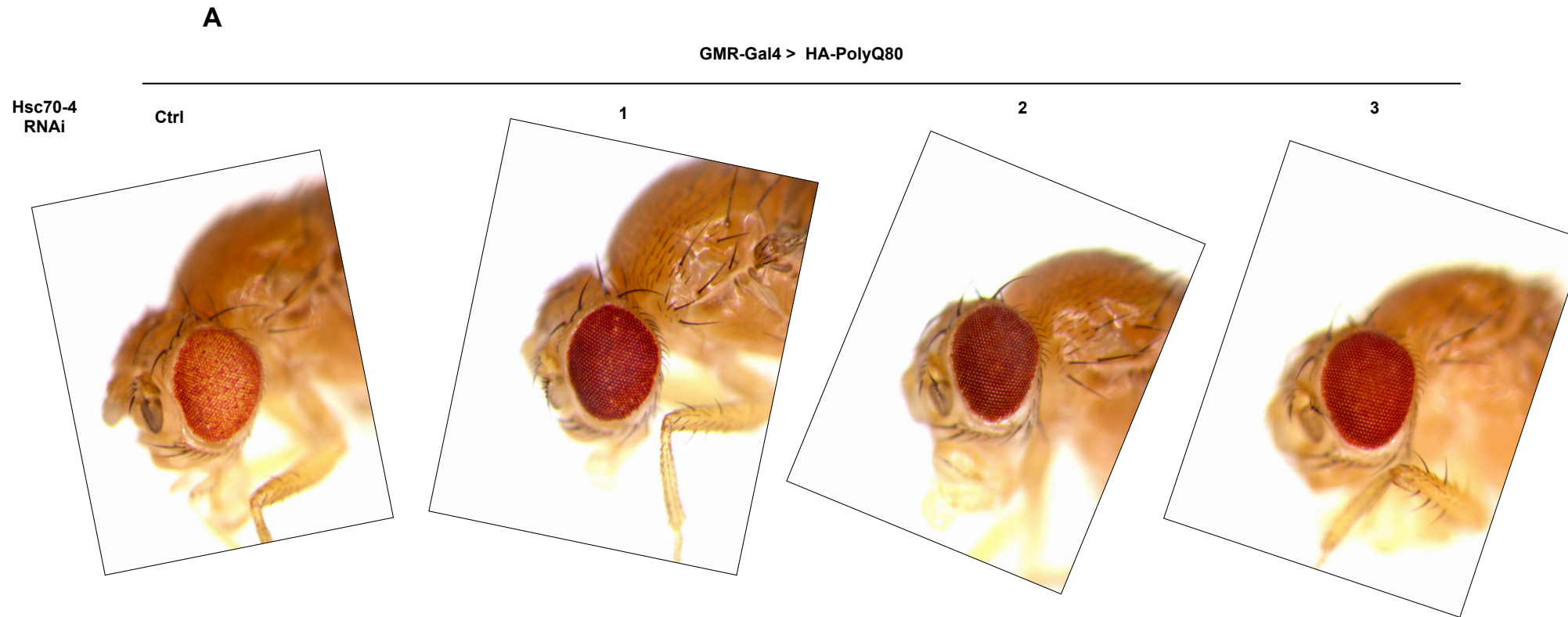

Figure 7B

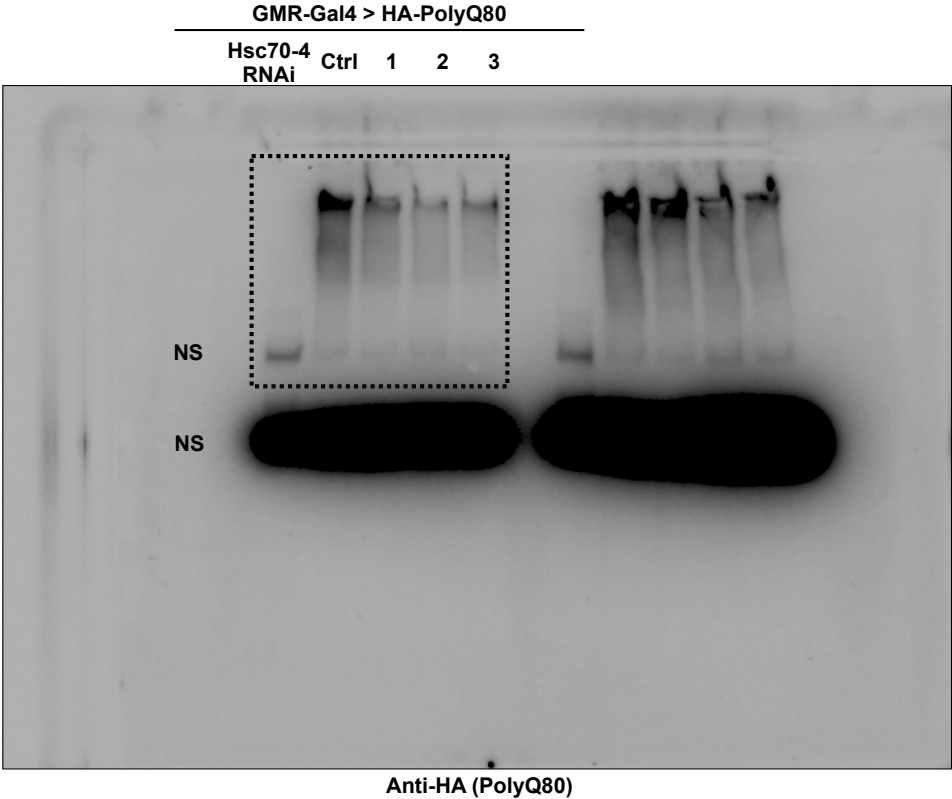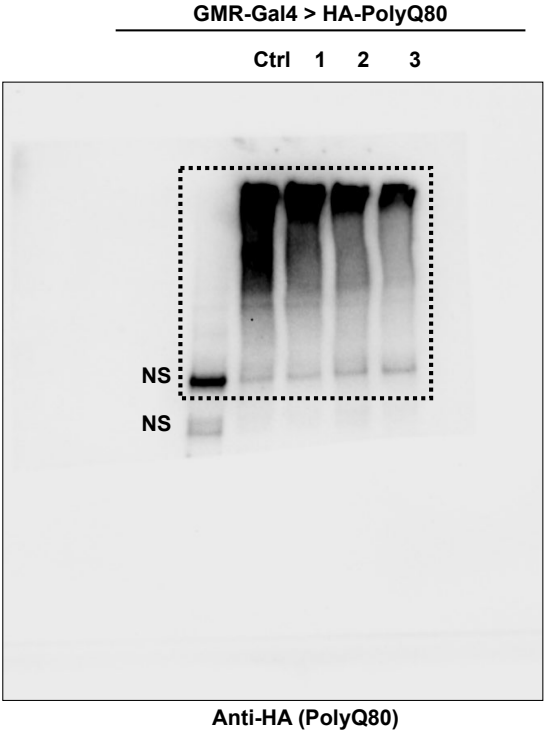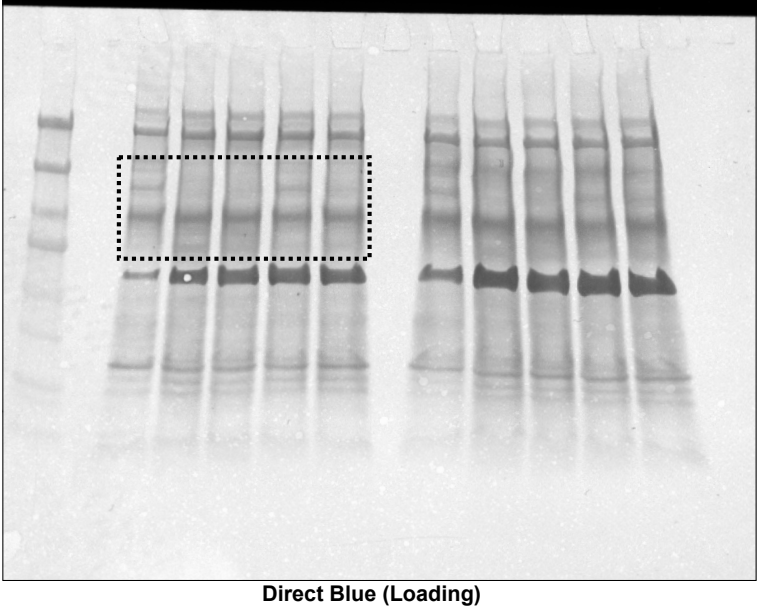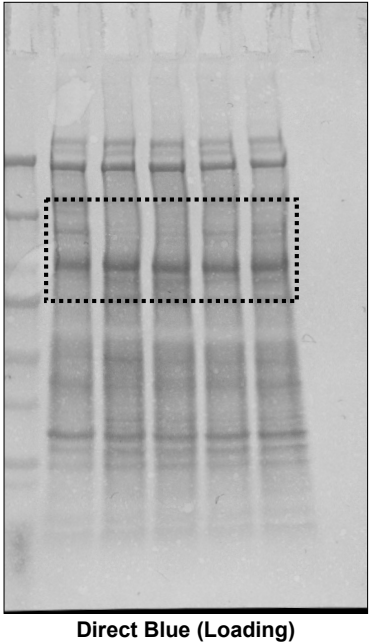

**Figure 7C**

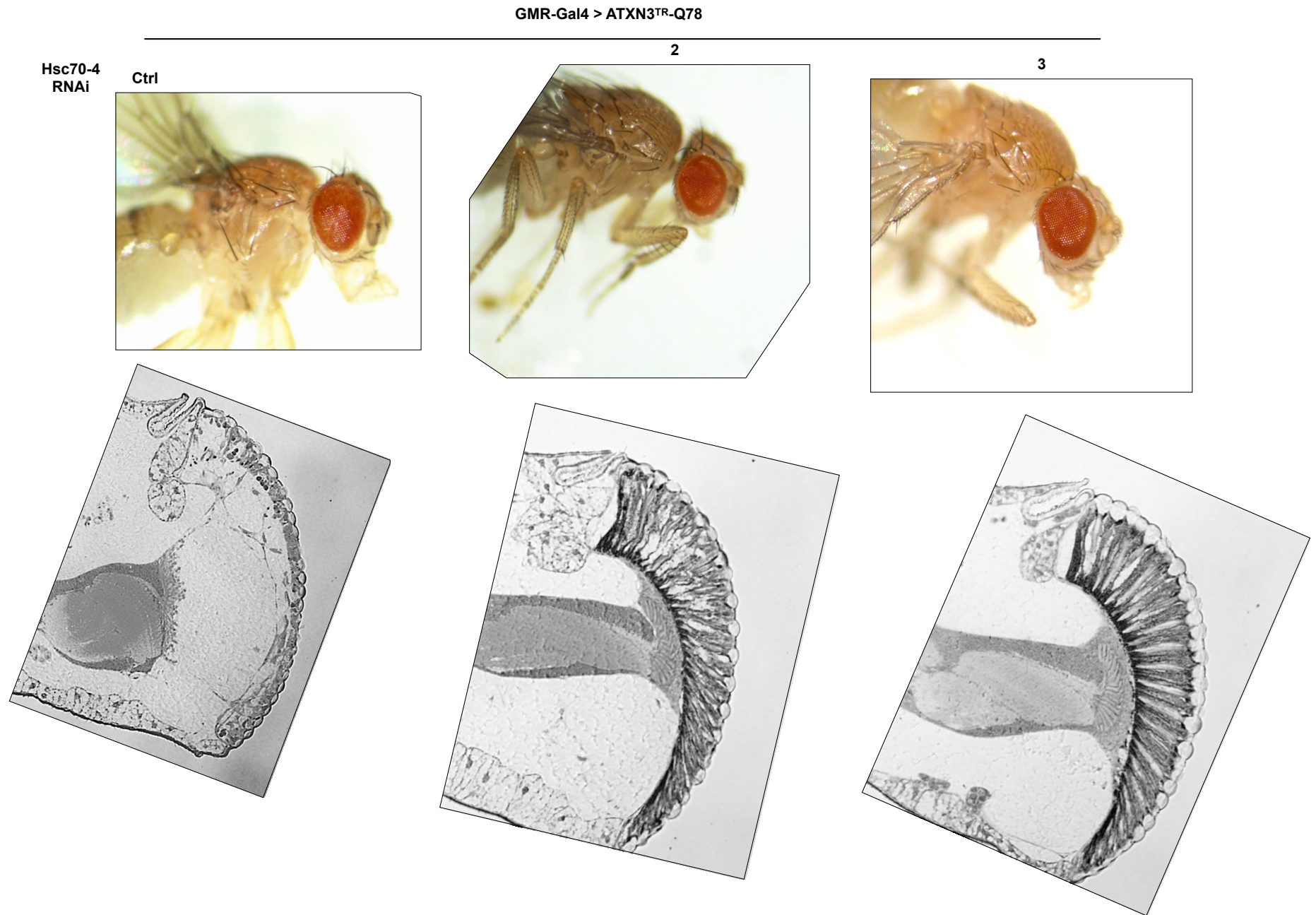

Supplement: Figure 7—source data 1. [file elife-60742-fig7-data1.pdf.zip › Figure7SourceData.pdf]
