# Supplementary material for: Ubiquitin-interacting motifs of ataxin-3 regulate its polyglutamine toxicity through Hsc70-4-dependent aggregation
Source: eLife. 2020 Sep 21;9:e60742. doi: 10.7554/eLife.60742 (PMC7505662; doi:10.7554/eLife.60742)
Supplement: Figure 8—source data 1. [file elife-60742-fig8-data1.pdf.zip › Figure8SourceData.pdf]

Figure 8A

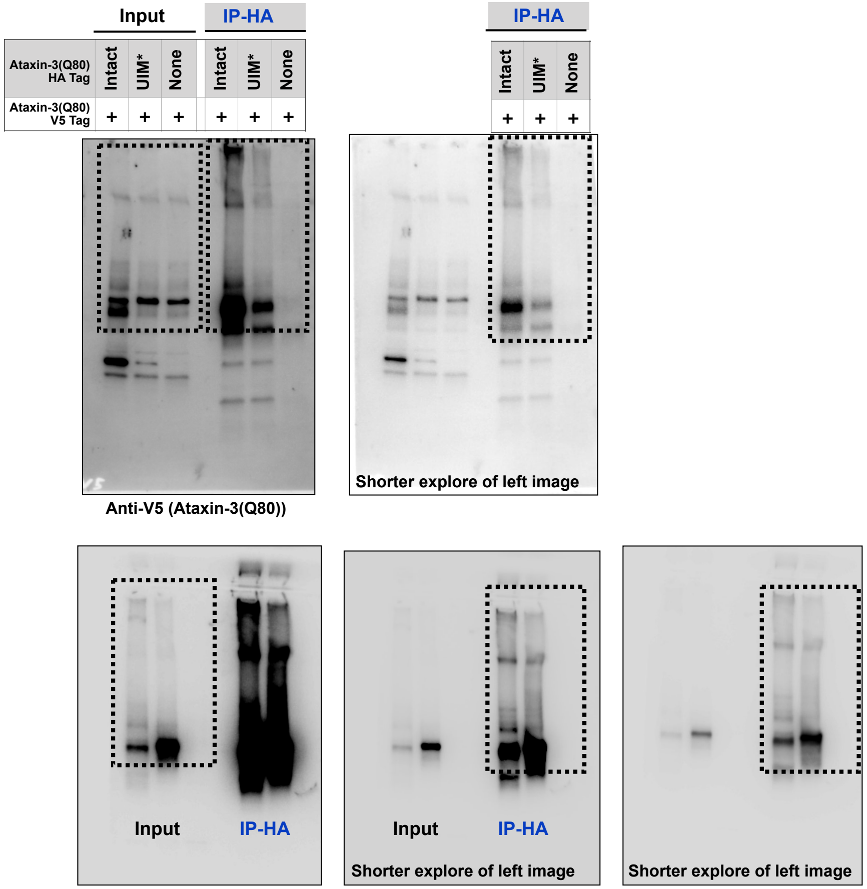

Anti-V5 (Ataxin-3(Q80))

Anti-HA (Ataxin-3(Q80))

Figure 8B

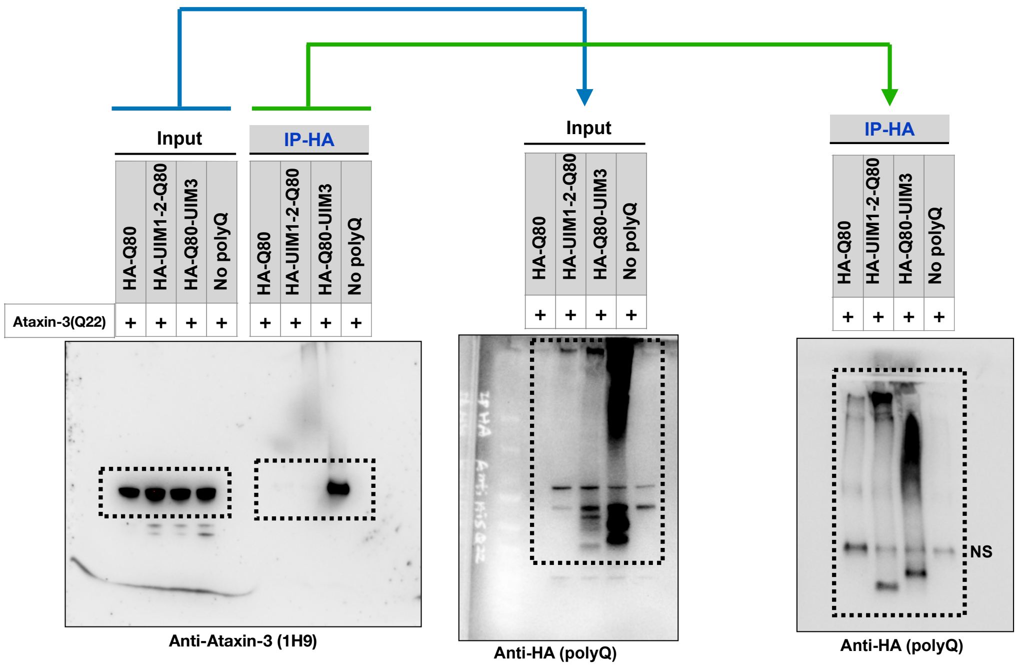

Due to persistent antibody signal and imaging issues after stripping, the same samples and quantities were loaded on different membranes and probed with the specified antibodies.
